# Ophthalmic manifestations of novel coronaviruses: precautionary measures and diagnostic possibilities

**DOI:** 10.7189/jogh.10.010340

**Published:** 2020-06

**Authors:** Suzana Konjevoda, Samir Canovic, Zrinjka Pastar, Irena Tabain, Vladimir Savic, Ljubo Barbic, Boris Dzelalija, Katarina Vukojevic, Vladimir Stevanovic, Snjezana Mardesic, Ivona Kosovic, Tatjana Vilibic-Cavlek

**Affiliations:** 1 General Hospital Zadar, Zadar, Croatia; 2 University of Zadar, Zadar, Croatia; 3 Croatian Institute of Public Health, Zagreb, Croatia; 4 Croatian Veterinary Institute, Zagreb, Croatia; 5 Faculty of Veterinary Medicine University of Zagreb, Zagreb, Croatia; 6 University of Split School of Medicine, Split, Croatia; 7 School of Medicine, University of Zagreb, Zagreb, Croatia

Coronaviruses (CoVs) are diverse group of viruses that mainly affect birds and mammals. In contrast to common CoVs, novel CoVs, such as severe acute respiratory syndrome coronavirus (SARS-CoV), Middle East respiratory syndrome coronavirus (MERS-CoV) and SARS-CoV-2 are highly pathogenic, especially in the elderly and immunocompromised patients [1]. CoV are transmitted mainly through respiratory droplets and contact with infected persons, however the other routes of transmission including feco-oral as well as via contaminated hands or surfaces remain to be further studied [2].

Since reports suggest that number of patients presented with extra-pulmonary manifestations, including conjunctivitis, it is possible that SARS-CoV-2 could be transmitted by aerosol contact with conjunctiva as has been shown for SARS-CoV [3-6]. Recent reports indicated that many ophthalmologists involved in the diagnosis and treatment of the COVID-19 accidentally acquired SARS-CoV-2 infection, even from asymptomatic patients [7]. For example, according to data of the American Academy of Ophthalmology, an ophthalmologist working in Wuhan contracted the virus from an asymptomatic glaucoma patient in January 2020. Ophthalmologists may be particularly susceptible to infection due to the proximity between the patient and ophthalmologist during the ophthalmoscopy slit-lamp microscope examination which may be a source of spread among health care workers [8].

Rapid collection and testing of appropriate specimens from suspected COVID-19 patients is a priority for clinical management and outbreak control [9]. Real-time reverse-transcriptase polymerase chain reaction (RT-PCR) is recommended for diagnosis of SARS-CoV-2 infection. The viral loads in throat swab and sputum samples peaked at around 5-6 days after disease onset, ranging from 10^4^ to 10^7^ copies/mL during this time. Sputum samples generally showed higher viral loads than throat swab samples [9,10].

Several recently published studies analyzed the presence of COVs in ocular samples (**Table 1**). In a study conducted in 2003 (Hong Kong) among 20 patients with SARS-CoV, tear swabs and conjunctival scrapings were taken randomly from one eye of all recruited patients and analyzed by culture and RT-PCR. SARS-CoV was not isolated in viral culture or detected by RT-PCR neither in tear nor in conjunctival scraping samples. Although the presence of virus cannot be excluded with certainty due to possible false-negative results, it seemed that conjunctival swabs or conjunctival scrapings are not useful samples for confirming or excluding the SARS-CoV diagnosis [11]. However, in another case series from Singapore, SARS-CoV was detected in tears of 3/36 tested patients sampled in the early phase (within 9 days of onset) of disease. This was the first case series reported with the detection of the SARS-CoV from tears and has important implications for the ophthalmology practice [12].

**Table 1:** Detection of novel coronaviruses in ocular samples

Year/Place	Virus	Clinical samples	Method	Tested patients	Positive patients	Reference
2003/Hong Kong	SARS-CoV	Tear swabs; conjunctival scrappings	RT-PCR; virus isolation	20	0	[11]
2003/Singapore	SARS-CoV	Tears	RT-PCR	36	3	[12]
2019-2020/Wuhan	SARS-CoV-2	Ocular discharges	RT-PCR	72	1	[4]
2020/Hangzhan	SARS-CoV-2	Tears; conjunctival secretions	RT-PCR	30	1	[3]
2020/Wuhan	SARS-CoV-2	Conjunctival swabs	RT-PCR	67	1/2*	[5]

RT-PCR – real-time reverse-transcriptase polymerase chain reaction

*Probable positive.

Three recent studies from China (one published and two non peer-reviewed preprints) assessed the tears and conjunctival secretions in SARS-CoV-2-infected patients. In a study conducted in Hangzhou on 30 patients, samples of tear and conjunctival secretions obtained from the only one patient with conjunctivitis yielded positive RT-PCR results [3]. One study from Wuhan identified conjunctivitis in two (2.78%) of 72 patients with confirmed COVID-19 infection. SARS-CoV-2 RNA was found in ocular discharges by RT-PCR in one patient. Although SARS-CoV-2 was rarely detected in ocular samples, the possibility of eye infection and the ocular route as a potential infection source should be considered and further examined [4].

Another study from Wuhan enrolled 67 cases with confirmed or probable COVID-19 infection. Conjunctival swab samples from one patient showed positive RT-PCR result, while two patients yielded probable positive RT-PCR result. None of the patients showed ocular symptoms. The only one patient with conjunctivitis as the first symptom had negative conjunctival sac test for SARS-CoV-2. Although the authors suspected the incidence of SARS-CoV-2 infection through conjunctiva is extremely low, the nosocomial infection after occupational exposure is a possible route [5]. However, all these results are based on a small number of patients, therefore further studies are needed to confirm this observation and evaluate the impact of SARS-CoV-2 on eyes [3-5].

In contrast to human CoVs, nonhuman CoVs frequently cause ocular disease in different animal species following intraocular inoculation which indicate that ocular exposure may represent a meaningful route of entry for these viruses and should not be ignored [1,13].

**Figure:**
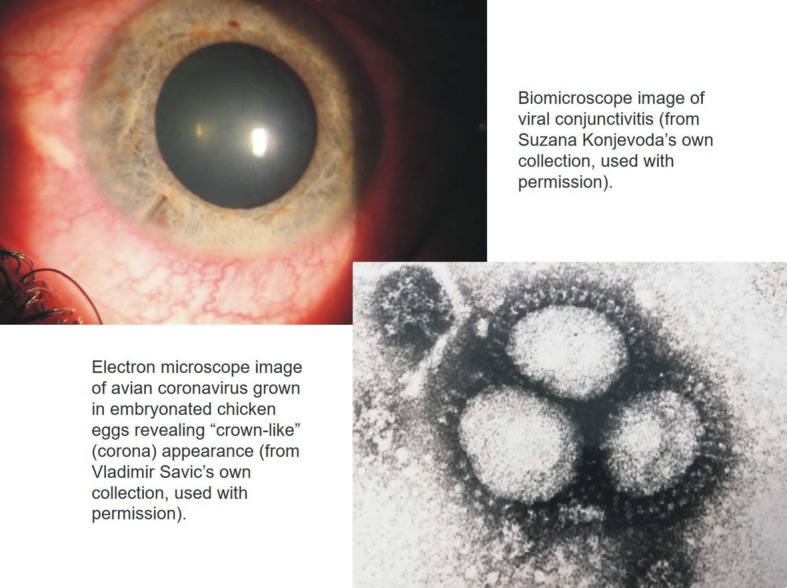
Photo: From co-author's own collections (used with permission).

Recent data showed that novel CoVs can remain infectious on inanimate surfaces (metal, glass, plastic) at room temperature for up to 9 days, while higher temperatures (>30°C) reduced the duration of virus persistence [14]. In addition, one study evaluated the stability of SARS-CoV-1 and SARS-CoV-2 in aerosols. SARS-CoV-2 remained viable in aerosols throughout the duration of experiment (3 hours), with a reduction in infectious titer from 10^3.5^ to 10^2.7^ TCID_50_ per liter of air [15]. CoVs can be efficiently inactivated by surface disinfection procedures with 62%-71% ethanol, 0.5% hydrogen peroxide or 0.1% sodium hypochlorite within 1-minute exposure. Other biocidal agents such as 0.05%-0.2% benzalkonium chloride or 0.02% chlorhexidine digluconate are less effective. A similar effect can be expected against the SARS-CoV-2 [11].

## CONCLUSION

Studies so far have shown multiple routes of CoVs transmission including conjunctiva. The presence of CoVs in tears and conjunctival discharges may affect precaution practices and sites of sampling for CoVs diagnosis. These secretions may be potentially hazardous, however, there are still no data about the infectivity (viral load) and clinical significance of these specimens. Although contamination from the upper respiratory tract cannot be ruled out, the ability to detect and isolate the SARS-CoV-2 in tears in some patients should be considered as an additional diagnostic tool since tear sampling is both simple and easily repeatable.

Although conjunctivitis is not common symptoms of SARS-CoV-2, we would like to point out that ophthalmologists should keep in mind that patients from areas with documented virus circulation may represent cases of COVID-19, therefore, it is very important to recognize possible early ocular manifestations [15]. The presence of SARS-CoV-2 in ocular discharges emphasizes the need for appropriate precaution to prevent transmission through ocular secretions [16]. Protection for the mouth, nose (N-95 mask) and eyes (goggles or shield) when managing patients potentially infected with SARS-CoV-2 should be recommended in order to reduce the risk of infection and increase the control of disease.

In addition, although the viral load of novel CoVs on inanimate surfaces is not known, it is expected that surface disinfection with commonly used disinfectants should be effective in reducing the virus transmission. Moreover, ophthalmic instruments, especially those in direct contact with patient's mucosal membranes should be immediately disinfected. Frequent hand washing is also very important.
